# Multiple Plantar Poromas in a Stem Cell Transplant Patient

**DOI:** 10.7759/cureus.8773

**Published:** 2020-06-22

**Authors:** Rachel L Marsh, Benjamin Kaffenberger, Llana Pootrakul, Catherine Chung

**Affiliations:** 1 Dermatology, The Ohio State University College of Medicine, Columbus, USA; 2 Dermatology, The Ohio State University Wexner Medical Center, Columbus, USA; 3 Division of Dermatology and Dermatopathology, The Ohio State University Wexner Medical Center, Columbus, USA

**Keywords:** eccrine poroma, poromatosis, multiple poromas, stem cell transplantation, acute myeloid leukemia, lymphoproliferative neoplasm, graft versus host disease, chronic immunosuppression

## Abstract

Poromatosis, or the formation of multiple eccrine poromas, is associated with chronic immunosuppression, lymphoproliferative neoplasms, and stem cell transplantation, though the etiology and clinical significance remain poorly understood. Eccrine poromas are asymptomatic, may appear years after treatment, and overlap morphologically with other diagnoses, particularly human papillomavirus-associated verrucae, to which immunosuppressed patients may be predisposed and commonly occur in similar sites. We report a 47-year-old female on chronic immunosuppression who developed multiple plantar eccrine poromas three years after achieving acute myeloid leukemia (AML) remission following treatment with chemotherapy, total body irradiation, and allogenic stem cell transplantation. We propose that early recognition, timely treatment, and regular follow-up skin examinations are necessary in the setting of multiple poromas to reduce the risk of malignancy and avoid delays in diagnosis.

## Introduction

Poromas, benign adnexal tumors composed of apical glandular ductal cells with presumed eccrine origin, are commonly present in middle-aged adults as asymptomatic slow-growing solitary lesions. They are characterized by smooth or verrucous flesh-colored papules, nodules, or plaques with acral predilection, presumably due to high eccrine sweat gland density [1]. However, recent evidence suggests the scalp, trunk, and extremities are involved more frequently than previously believed [2]. Diagnostic histologic features include broad anastomosing bands of cuboidal-shaped epithelial cells with monomorphous nuclei and minimal cytologic atypia [3]. Rarely, patients develop grouped or widespread multiple eccrine poromas, known as eccrine poromatosis. Poromatosis has previously been associated with lymphoproliferative neoplasms and stem cell transplantation, though the etiology and clinical significance remain poorly understood. This report adds to the limited literature of poromatosis in transplant patients with a history of hematologic malignancy. 

## Case presentation

**Figure 1:**
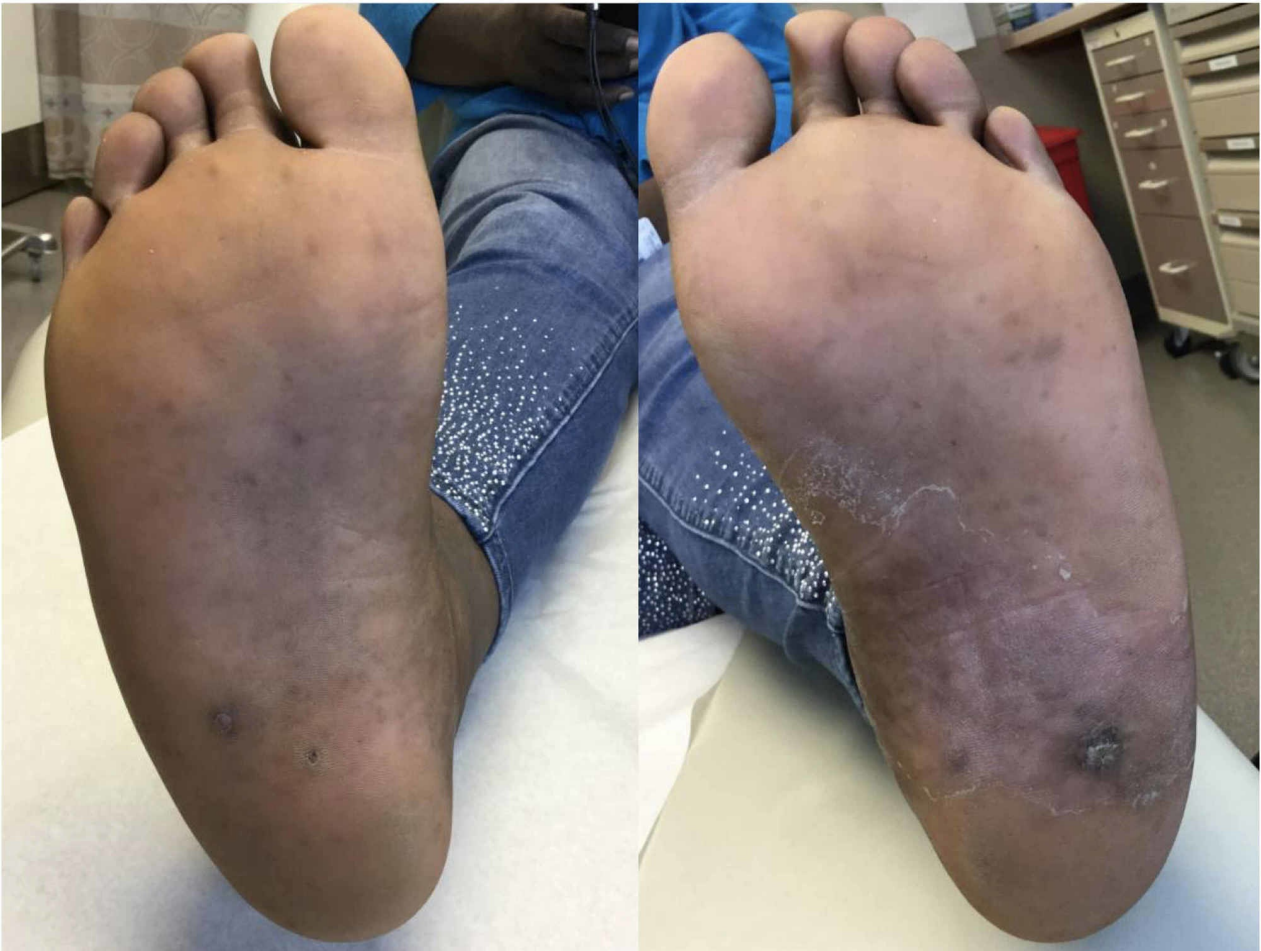
Multiple verrucous and hyperkeratotic lesions on the bilateral plantar feet.

A 47-year-old African American female presented with a rapidly growing, tender lesion on the left plantar foot. The patient was diagnosed with acute myeloid leukemia (AML) three years prior to presentation and was treated with high dose cytarabine, daunorubicin, intrathecal methotrexate, and total body irradiation followed by an allogenic stem cell transplant. She achieved remission, but her post-transplantation course was complicated by multisystem graft-versus-host disease (GVHD) requiring long-term immunosuppressive therapy. Examination of the feet revealed a 1 cm verrucous plaque on the left plantar surface with an adjacent 0.5 cm papule and two similar-appearing lesions on the right plantar foot (Figure 1). 

**Figure 2:**
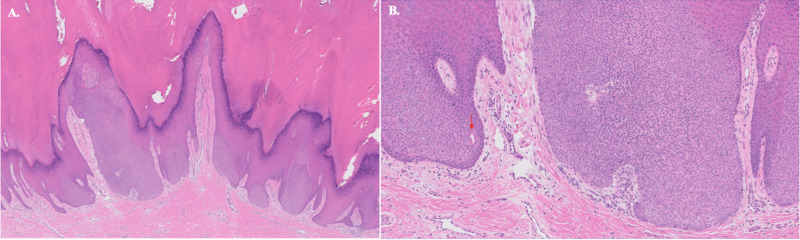
Histopathologic findings of plantar eccrine poroma. A. Low-power magnification demonstrates papillomatosis, hyperkeratosis, and an intraepidermal proliferation of pale-staining squamoid cells (H&E 5x).  B. Higher magnification reveals a monomorphous proliferation of ovoid cells with duct formation (arrow; H&E 20x). All lesions sampled demonstrated similar histopathologic findings.

The clinical impression was verruca, but given the patient’s immunocompromised status, shave biopsy was performed on the left plantar foot to exclude malignancy. The lesion demonstrated epidermal papillomatosis with hyperkeratosis and intraepidermal proliferation of monomorphous ovoid cells with formation of ductal spaces, consistent with intraepidermal poroma (Figure 2). Excisional biopsies were then performed on remaining lesions, demonstrating similar histopathologic findings. Lesions did not recur after 15 months of serial follow-up skin examinations.

## Discussion

Multiple poromas have been reported in patients with a history of lymphoproliferative neoplastic conditions treated with radiation therapy, chemotherapy, and stem cell transplantation [4-12]. Importantly, onset of multiple poromas involving acral and nonacral sites can vary from months to years after treatment cessation. Because solitary poromas can develop at sites of trauma, it is hypothesized radiotherapy and chemotherapy may facilitate poromatosis through cytotoxic-mediated damage and subclinical injury in the eccrine glands [8]. Remodeling or regeneration of damaged eccrine apparatuses may explain long latency periods between treatment cessation and the development of multiple poromas [5]. Chronically immunosuppressed patients appear at higher risk for poromatosis. There are several reports of multiple poromas developing in patients with GVHD on chronic immunosuppression following allogenic stem cell or bone marrow transplants [4, 7]. Human papillomavirus (HPV) infection in immunosuppressed patients may further potentiate the development of multiple eccrine poromas [4].

Data on longitudinal outcomes and evidence-based treatment guidelines are lacking in patients with multiple poromas. Solitary poromas carry a favorable prognosis, and treatment with surgical excision or electrosurgical destruction is curative. However, it is estimated that 18% of longstanding solitary eccrine poromas may progressively degenerate into malignant porocarcinoma [13]. Porocarcinomas have a propensity to metastasize through lymphatic spread, and thus require prompt treatment with wide local excision or Mohs micrographic surgery. Individuals with a history of immunosuppression, exposure to chemical agents, and chronic ultraviolet exposure are at increased risk for porocarcinoma development [14]. These risk factors overlap with factors associated with eccrine poromatosis, suggesting patients with multiple poromas may have a higher risk of developing porocarcinoma. However, there remains a paucity of data on malignant transformation rates and de novo porocarcinoma formation in the context of eccrine poromatosis. While more data are needed to elucidate long-term clinical outcomes, we propose that serial follow-up full body skin examinations are necessary to mitigate risk of malignancy in patients with poromatosis.

## Conclusions

In summary, we report a 47-year-old female in AML remission on chronic immunosuppressive therapy who developed multiple plantar eccrine poromas. Eccrine poromas overlap morphologically with HPV-associated verrucae, to which these patients may be predisposed and commonly occur in similar sites. To facilitate early recognition and avoid delays in diagnosis, clinicians should consider eccrine poromas in the context of pertinent history and physical exam findings. Further study is needed to clarify mechanisms facilitating poromatosis and enhance guidelines for appropriate therapeutic management.
